# Engineering Iron Oxide Nanocatalysts by a Microwave-Assisted Polyol Method for the Magnetically Induced Degradation of Organic Pollutants

**DOI:** 10.3390/nano11041052

**Published:** 2021-04-20

**Authors:** Alvaro Gallo-Cordova, Sabino Veintemillas-Verdaguer, Pedro Tartaj, Eva Mazarío, María del Puerto Morales, Jesús G. Ovejero

**Affiliations:** 1 Instituto de Ciencia de Materiales de Madrid, ICMM/CSIC, C/Sor Juana Inés de la Cruz 3, 28049 Madrid, Spain; sabino@icmm.csic.es (S.V.-V.); ptartaj@icmm.csic.es (P.T.); puerto@icmm.csic.es (M.P.M.); 2 Departamento de Química Física Aplicada, Facultad de Ciencias, Universidad Autónoma de Madrid, C/Francisco Tomás y Valiente, 7, Cantoblanco, 28049 Madrid, Spain; eva.mazario@uam.es

**Keywords:** iron oxide nanoparticles, microwave synthesis, magnetic induction heating, organic dyes, acid orange 8, methylene blue, adsorption, advanced oxidation process, wastewater

## Abstract

Advanced oxidation processes constitute a promising alternative for the treatment of wastewater containing organic pollutants. Still, the lack of cost-effective processes has hampered the widespread use of these methodologies. Iron oxide magnetic nanoparticles stand as a great alternative since they can be engineered by different reproducible and scalable methods. The present study consists of the synthesis of single-core and multicore magnetic iron oxide nanoparticles by the microwave-assisted polyol method and their use as self-heating catalysts for the degradation of an anionic (acid orange 8) and a cationic dye (methylene blue). Decolorization of these dyes was successfully improved by subjecting the catalyst to an alternating magnetic field (AMF, 16 kA/m, 200 kHz). The sudden temperature increase at the surface of the catalyst led to an intensification of 10% in the decolorization yields using 1 g/L of catalyst, 0.3 M H_2_O_2_ and 500 ppm of dye. Full decolorization was achieved at 90 °C, but iron leaching (40 ppm) was detected at this temperature leading to a homogeneous Fenton process. Multicore nanoparticles showed higher degradation rates and 100% efficiencies in four reusability cycles under the AMF. The improvement of this process with AMF is a step forward into more sustainable remediation techniques.

## 1. Introduction

Nanotechnology can be considered a “small” solution for many big problems, bringing novel benefits in terms of products and processes. Nowadays, there are already a wide range of studies on new nanomaterials that are transforming the available technology in areas as important as medicine (nanomedicine, theragnosis, etc.) and sustainable development (clean energy, environmental remediation, etc.) [1]. Among possible nanomaterials, magnetic iron oxide nanoparticles (MIONPs) stand out with their unique magnetic properties, low environmental impact, high biocompatibility, and wide versatility in technological fields such as wastewater treatment, catalysis, biomedicine, etc. [2,3]. This class of magnetic nanoparticles presents specific features like their large surface-to-volume ratio and high colloidal stability, a good magnetic response, and a powerful heating capacity under alternating magnetic fields [4].

During recent decades, MIONPs have been used for the removal and degradation of different contaminants in either waste or drinking water with outstanding results [5]. The increase of unmonitored pollutants with suspected human and ecological adverse effects has redirected the use of these nanoparticles towards the removal of hazardous organic compounds in the environmental area [6]. These contaminants can be derived from different sources like tanning, printing, paper production, food processing and pharmaceutical, being the textile industry the most important one. During the processing of fabrics and textiles, the dyeing process can generate large volumes of colored wastewater and different type of post-production wastes [7]. Within all these dyes, azo dyes can be linked to serious human health deterioration and comprise about two thirds of all synthetic dyes. These azo compounds are characterized by the presence of –N=N– groups in a typical R-N=N–R’ structure. Some examples of these azo dyes are methyl orange, alizarine yellow R, acid orange 5, and acid orange 8, among others [8]. Other common compounds are the synthetic basic dyes, such as methylene blue, that can also be used for medical purposes, creating a different source of pollution [9]. As these kinds of compounds are very stable to light and reluctant to conventional microbial treatment methods, it is important to find alternatives for their removal and degradation from water [7]. In this endeavor, it is important to develop engineered materials by rapid, scalable, and efficient methods to face this environmental hazard.

The most common processes for removal of organic dyes in the current state of the art are biological processes with microalgae, activated carbons, membrane bioreactors [10], enzymatic degradation [11], and physical chemical processes like adsorption [12] and advanced oxidation (ozonation, photocatalysis, electrochemically assisted) [13,14]. MIONPs have been used as highly efficient adsorbents of different dyes that can also degrade these organic molecules [15]. In addition, Fe^2+^ and/or Fe^3+^ surface ions can react with hydrogen peroxide to produce highly oxidative species for the degradation of organic molecules (Fenton-like reactions). Moreover, MIONPs under the action of an alternating magnetic field (AMF) can become “self-heating” catalysts for the degradation processes enhancement. This radio-wave magnetic heating process provides a more efficient and sustainable way to increase temperatures for the improvement of reaction yields [16].

Previous research already examined the ability of different shaped and structured MIONPs in the removal of organic pollutants as well as the adsorption of heavy metals present in drinking and wastewater [17,18,19]. However, there are few references that exploit the radio-wave magnetic heating-assisted degradation of this kind of pollutant. For example, it was successfully proven that the degradation of methylene blue can be enhanced by subjecting the MIONPs to an AMF [20], and analogous studies have showed the degradation of antibiotics in a much faster way under an AMF [21]. Self-heating magnetic materials have also been tested as an enabling technology in catalytic processes like hydrodeoxygenation of acetophenone derivates [22], water electrolysis [23], and CO_2_ methanation by the Sabatier reaction [24]. This pioneering work has proved that self-heating catalysts can be used in complex reactions performed at relatively high temperatures (>300 °C), but there are still a limited number of examples in which this technology is applied to water remediation.

Several synthesis methods have been studied to obtain MIONPs in a reproducible and scalable manner. Even though there is not a standard method, each technique has its own advantages and drawbacks. One interesting method, firstly described by Fievet et al. [25], is the polyol process in which ethylene glycol (or some derivate) acts as both solvent and surfactant, providing control over the nanoparticle growth and aggregation [26]. Polyols are low-molecular-weight molecules with high boiling points that increase the reflux temperature of the reaction just like the well-known organic thermal decomposition procedure with the advantage of obtaining particles that can be suspended in water without further processing [27]. A fundamental benefit of polyol solvents when compared to common organic solvents used in high-temperature decomposition is that polyol molecules are highly polar. Thus, it is possible to combine this polyol procedure with a more efficient heating technology such as the microwave-assisted (MW) technology, leading to higher production yields in shorter reaction times [28]. The MW heating offers an effective energy transference through the reaction solution with minimum temperature gradient in the reactor. The combination of small thermal gradient and vigorous stirring results in being crucial to establish homogeneous reaction conditions in the whole reactor and obtain a monodispersed sample. Therefore, the MW-assisted polyol procedure seems to be a great alternative for engineering efficiently well-dispersed and uniform MIONPs. In fact, the results in the MW synthesis of ferrite nanoparticles (MFe_2_O_4_, M: Fe, Co, Zn) confirmed such expectations [29]. MW heating is also quite convenient to reduce reaction times from days to hours or even a few minutes [30].

The main objective of this article is to engineer different MIONPs systems by the MW-assisted polyol method and test their magnetic heating efficiencies on the degradation of organic dyes under an alternating magnetic field. First, the key parameters for the preparation of single-core and multicore flower-like MIONPs were studied by optimizing this MW-assisted procedure in terms of solvent, water content, reaction time, and heating ramp. By adjusting the size and the aggregation state of the samples, it was possible to alter the magnetic response, and therefore also the efficiency as wastewater treatment agents. Secondly, the suitability of two selected nanostructures (single-core and flower-like MIONPs) in the adsorption and degradation of organic dyes was analyzed for conventional and radiowave-mediated heating. For this purpose, acid orange 8 (AO8) and methylene blue (MB) were used as model anionic and cationic dyes of interest in the textile industry. It should be considered that MIONPs under an alternating magnetic field can reach high surface temperatures in just seconds and heat up solutions from the inside, ensuring a better heat transfer and improving surface reactions. For this reason, the heating power of the samples prepared was also characterized in detail.

## 2. Materials and Methods

### 2.1. Chemical Reagents and Analysis

**Figure 1:**
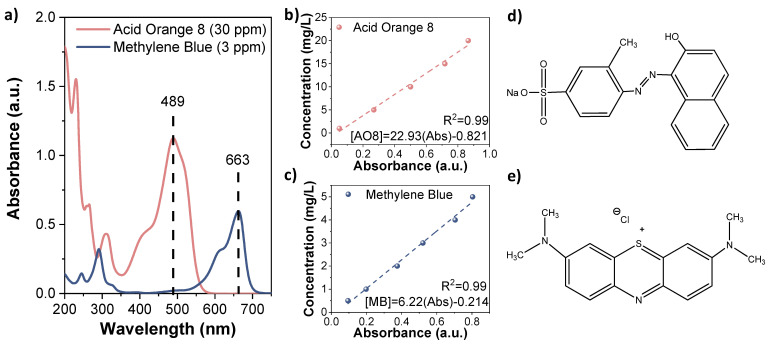
(**a**) UV/Visible spectra of acid orange 8 (AO8, 30 ppm) and methylene blue (MB, 3 ppm); (**b**,**c**) calibration curve, concentration (mg/L) vs. absorbance (a.u.) of AO8 and MB; (**d**,**e**) molecular structures of AO8 and MB.

Raw materials were purchased form Sigma Aldrich (San Luis, MO, USA): Iron(II) acetate (Fe(OAc)_2_, ≥99%), ethylene glycol (EG, ≥99.5%), diethylene glycol (DEG, 99%), triethylene glycol (TREG, 99%), tetraethylene glycol (TEG, ≥97%), hydrogen peroxide (H_2_O_2_, 35%), ethanol (99.8%), acid orange (AO8, 65%), methylene blue (MB), dimethyl sulfoxide (DMSO, for molecular biology), and benzoquinone (BQ, ≥99%). Colorimetric analyses were carried out to quantify the degradation yields of the organic dyes. UV/Visible spectrum for AO8 and MB, before and after treatments, was obtained using a Perkin-Elmer LAMBDA 35 UV–visible spectrophotometer (Waltham, MA, USA). Calibration curves as a function of the concentration at the maximum absorbance (489 and 663 nm for AO8 and MB, respectively, Figure 1a) were performed using a Biochrom WPA Biowave DNA UV-visible spectrophotometer (Cambridge, UK). and are presented in Figure 1b,c. The molecular structure of dyes is shown in Figure 1d,e.

### 2.2. Synthesis of Iron Oxide Nanoparticles

Single-core and multicore MIONPs were prepared by optimizing a previously described microwave-assisted polyol method for the synthesis of single-core ferrite nanoparticles [29]. The synthesis was carried out in a microwave oven Monowave 300^®^ (Anton Paar GmbH, Graz, Austria) working at 2.45 GHz. The reaction was monitored by a temperature controller and shaken by a built-in magnetic stirrer. Our initial conditions were based on parameters already optimized in a previous paper (water content 3.7%, heating ramp 3.75 °C/min, time 2 h and temperature 170 °C) [29]. Here, the influence of different polyol solvents (EG, DEG, TREG, and TEG), the water content (0 and 3.7%), the reaction temperature (170 and 220 °C), and the heating ramp (3.75, 7.4, 14.6 °C/min) are studied.

Two of the obtained samples, one a single core and the other multicore, were selected for the organic dyes removal and magnetic induction heating-assisted degradation. Following the standard procedure, samples are prepared as follows: 300 mg of Fe(OAc)_2_ were dissolved in 19 mL of a mixture polyol/water (96.3:3.7 *v*/*v*). For multicore flower-like particles (NF60), EG was used as a solvent, while DEG was used for single-core particles (NP15). A glass vial with the mixture was then placed in the microwave reactor with 600 rpm stirring and heated at 3.75 °C/min until 170 °C. The reaction was maintained at this temperature for 2 h and rapidly cooled down afterwards. The product was washed several times with ethanol and finally dispersed in distillate water using sonication (Elmasonic S30 ^®^ from Elma Schmidbauer GmbH, Singen, Germany).

### 2.3. Characterization of Iron Oxide Nanoparticles

Structural characterization of the samples was carried out by transmission electron microscopy, X-ray diffraction, thermogravimetric analysis, infrared spectroscopy, and elemental analysis. A transmission electron microscopy with JEOL JEM 1010 at 100 keV was used to determine the mean particle size and distribution. Sample preparation consists of the deposition of a drop of diluted particles in a carbon-coated copper grid, which will eventually evaporate at room temperature. Around 200 particles were measured on different micrographs and the particle size data were fitted to a lognormal distribution. The colloidal properties of the MIONPs were analyzed by dynamic light scattering (photo correlation spectroscopy) with a Malvern Instrument Zetasizer Nano SZ (Malvern, UK) equipped with a solid-state He-Ne laser (λ = 633 nm). In order to determine iron oxide phase and crystal size, X-Ray diffraction (XRD) was recorded between 20° and 70° (2Ɵ) in a diffractometer with a graphite monochromator (Bruker D8 Advance, Billerica, MA, USA) and CuKα radiation (k = 1.5406 Å). Mean crystal size was estimated from (311) reflection using Scherrer’s equation. To quantify the organic content, thermal analysis (TGA) of the MIONPS was performed using a TA Instrument Q2000 calorimeter (New Castle, DE, USA), heating up to 800 °C at 10 °C/min in air and measuring the weight loss. To detect the rest of solvent on the MIONPs surface, Fourier-transformed infrared spectra between 400 and 4000 cm^−1^ were recorded using a Bruker IFS 66VS. The powdered samples were mixed and pressed into pellets in KBr at 2% *w*/*w*. Finally, inductively coupled plasma optical emission spectroscopy (ICP-OES) with a Perkin Elmer apparatus (OPTIME 2100DV, Waltham, MA, USA) was used to measure the iron concentration in the final colloids and the iron leaching after the Fenton process.

Static and dynamic magnetic characterization was carried out in a vibrating sample magnetometer (MagLabVSM, 9T, Oxford Instrument, Abingdon, UK) and in a Fives Celes AC field generator (model N° 12118 M01, Lautenbach, France), respectively. Magnetization curves were measured at room temperature under a maximum magnetic field of ±2400 kA/m. Powdered samples were pressed into sample holders and hysteretic parameters such as saturation magnetization (Ms), remanent magnetization (Mr), and coercivity (Hc) were obtained. Specific Absorption Rate (SAR) was measured in a hyperthermia apparatus equipped with a water-cooled, 6-turns, copper coil of 50 mm diameter, an insulation chamber at 21 °C, and a fiber optic temperature sensor OSENSA’s FTX. At least 500 µL of suspension at 1 g/L was placed in the center of the heating coil, and field frequencies (100–200 kHz) and amplitudes (8–40 kA/m) were varied.

Finally, the N_2_ adsorption-desorption isotherms were recorded using TriStar II 320 (Micromeritics, Norcross, Georgia) equipment at 77 K with different partial pressures on previously degasified (393 K, 0.1 mbar, 12 h) samples of MIONPs. The specific surface area and average pore diameter were calculated using BET equation and BJH method.

### 2.4. Adsorption Experimentation

The adsorption process was analyzed for single-core and multicore particles. Samples consisting of 10 mg MIONPs dispersed in 10 mL organic dye solution (500 ppm) were mechanically shaken, and aliquots were taken at different times. Once equilibrium was reached, magnetic nanoparticles were quickly separated from the media (<1 min) using an external NdFeB magnet (320 kA/m field gradient). The final concentration of the organic dye solution after the adsorption process was determined by colorimetric analyses and the adsorption capacity was evaluated. Parameters like effect of pH and adsorption time (5, 15, 30, 60, 120 min) were considered in the study.

### 2.5. Thermal and Magnetic Induction Heating-Assisted Degradation

The degradation of the organic compounds was analyzed for single-core and multicore nanoparticles by following the decolorization of the dye’s samples. The kinetic of the process was measured in the dark at 25 °C and 90 °C in a thermal reactor (Eppendorf thermomixer comfort, Hamburg, Germany) and under magnetic induction heating (200 kHz, 16 kA/m) using the same equipment as for SAR measurement.

The thermal heating-assisted degradation experimentation consisted of mixing the MIONPs (1 g_MIONPs_/L in the final mixture) with 50 mL of the AO8 or MB aqueous solution (500 mg/L) at pH 3.5. These high pollutant concentrations were selected as industrial wastewaters usually present values of 500 mg/L of chemical oxygen demand [31]. Prior to starting the Fenton process, the MIONPs were exposed to the dyes adsorption until equilibrium was reached, at 25 °C and 90 °C. Then, to start the organic compounds degradation, the optimum H_2_O_2_ (0.3 M) was added. The decolorization kinetic was followed by taking sample aliquots of 2.5 mL at different times. The MIONPs were recovered by magnetic harvesting and the AO8 and MB concentration in the supernatant was colorimetrically determined.

The magnetic induction heating was carried out considering optimum AMF parameters (200kHz, 16 kA/m). Two experiments were performed: 1) Starting at room temperature and applying the AMF together with the addition of hydrogen peroxide, and 2) letting the solution reach 90 ºC before adding hydrogen peroxide. In the second experiment, the concentration of particles was settled to 2 and 3 g_MIONPs_/L for NP15 and NF60, respectively, to reach such temperature. In all cases, the adsorption equilibrium was reached at 25 ºC and 90 ºC respectively prior the addition of H_2_O_2_.

Decolorization yield (DY) was calculated by using DY = 100 − 100C_2h_/C_0_ where C_0_ and C_2h_ are the initial dye concentration and the concentration after 2 h, respectively. The obtained data were fitted to a pseudo-first-order model to determine the degradation rate. Reusability tests were performed using NF60 (3 g/L) to degrade AO8 (500 ppm) under an AMF of 16 kA/m and 200 kHz frequency at 90 °C. Degradation cycles were performed by measuring the decolorization yield after 1 h of the H_2_O_2_ (0.6 M) addition in excess. In each cycle, an aliquot of the sample was extracted for analysis and an aliquot of the same volume of a AO8 mother solution of 5000 ppm was added to maintain the same proportions of NF60:AO8. This procedure was performed 4 times, and the efficiency of the process was estimated.

Finally, to elucidate the role of reactive oxygen species (ROS) on the oxidative degradation of AO8 and MB, the process was carried out in the presence of BQ and DMSO, used as ROS scavengers. The effect of these two scavengers on the degradation of AO8 and MB using NP15 and NF60 was performed under the following experimental conditions: 500 ppm of MB or AO8, 1 g/L of MIONPs, pH 3.5, H_2_O_2_ 0.3 M, 2 h, room temperature.

## 3. Results

### 3.1. Microwave-Assisted Synthesis

The microwave-assisted synthesis of MIONPs in polyol media has been explored for the preparation of single-core and multicore iron oxide nanoparticles. Some experimental parameters (iron concentration and reaction time) were kept constant as they were already optimized in previous works [28,29]. First, the effect of the heating rate and the water content using DEG as standard polyol solvent was explored. The data presented in Table 1 show that increasing the heating ramp from 3.75 up to 14.6 °C/min leads to a slight increase in particle size from 5.8 (± 1.0) to 7 (± 1) nm (Samples Ramp1, Ramp2, Ramp3, see Figure S1 for TEM images). To study the effect of water addition, the heating rate settled on 3.75 °C/min. It was observed that by adding 3.7% of water to the initial solvent mixture, it was possible to obtain well-dispersed 15 nm particles (NP15). Increasing the water content is a simple but straightforward way to reduce the boiling points of the polyols. In the case of EG, for example, it was found that by adding just 3.7% of water, the boiling point decreases from 197 °C to 170 °C without interfering with its reducing power. With the optimum heating ramp (3.75 °C/min), boiling point (170 °C), and water content (3.7%) fixed, the polyol length was varied to evaluate the effect on the particle size or aggregation.

Four different solvents were chosen with decreasing dielectric constants from EG to DEG, TEG, and TREG [32]. The dielectric constant is a good indicator of the sensitivity of the material to an external electric field at a certain frequency. As it can be seen in Table 1, the length of the polyol is critical for the size and geometry of the MIONPs synthesized. The final particle size obtained with polyols of longer chains than DEG was in all cases smaller than 5 nm under the same reaction conditions. These solvents with highly reducing power and lower dielectric constants, TEG and TREG, led to a rapid nucleation, consuming the Fe precursors for the subsequent growth (Samples solv1 and solv2). Whereas the particles obtained in EG, the solvent with the shortest polyol molecule, consist of multicore nanoparticles of 60 nm (± 8) mean diameter. These kinds of structures have been previously obtained in solvothermal synthesis using the same solvent but with different iron precursors [33]. The formation of nanoparticles can take place by nucleation and growth of initial nuclei or by assembly of primary nuclei forming the final single or multicore particle, respectively. The forces and the mechanism involved in these pathways are still poorly understood [34]. In general, it is the balance between electrostatic and steric forces that determines the formation of a single-core or a multicore structure. EG and DEG present different boiling temperatures, different dielectric constant (ε_r_(EG): 37; ε_r_(DEG): 32; at 20 °C, different viscosity, and chelating properties. These features will strongly affect the nuclei growth rate and they will determine the final colloidal assembly process [35]. With DEG having a longer chain, a better steric repulsion is expected between the initial nuclei, favoring the formation of single-core particles in the conditions used in this work (NP15). On the contrary, EG with a higher polarity led to a massive nucleation, forming small cores (7 nm) with weaker steric repulsion that aggregate to form the final multicore structure (NF60).

To sum up, the growth of the nanocrystals mediated by MW irradiation heating is heavily dependent on the solvents and may lead to a variety of sizes and morphologies. Water is considered as a first option due to its very high dipole moment and eco-friendliness, but in most cases, its use results in particle sizes below 10 nm and a poor heating power under AMF [36]. On the other hand, polyols solvents also present a high dipole moment and thus high absorption efficiency. However, in contrast to water, they can act as a particle’s surfactant, conferring a good colloidal stability to the products with minimal adverse effects to the environment [26].

Samples NP15 and NF60 were selected as interesting candidates for their further application in water remediation because of their specific microstructure and magnetic response. In the next section, the efficiency of 60 nm flower-like particles will be compared with 15 nm single-core particles in the adsorption and degradation of hazardous organic molecules such as AO8 and MB.

### 3.2. Iron Oxide Nanoparticles Characterization

**Figure 2:**
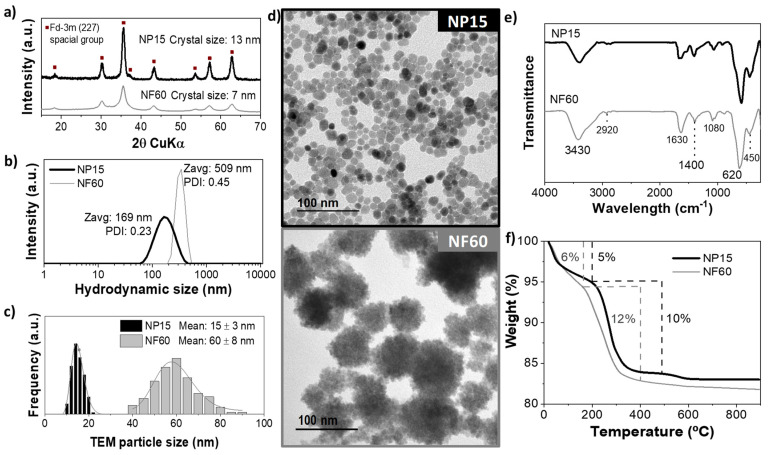
Iron oxide nanoparticles (NP15 and NF60) characterization. (**a**) X-ray diffractogram, (**b**) hydrodynamic size, (**c**) particle size distribution and log-normal fitting, (**d**) TEM images, (**e**) FTIR transmittance spectra, and (**f**) thermogravimetric analysis.

Figure 2 shows the characterization of some selected samples prepared by the MW-assisted polyol method. The crystalline character of the powder samples was confirmed by X-Ray diffraction. Cubic spinel iron oxide (Figure 2a) was identified as the only phase present in both cases. The (311) reflection was used to determine the crystal size of the samples using the Scherrer’s equation. A mean crystal size of 13 nm was obtained for NP15 and 7 nm for NF60. However, TEM mean size and distribution (Figure 2c) were 15 ± 3 and 60 ± 8 nm for NP15 and NF60, respectively. The discrepancies between TEM and X-ray size for NF60 confirm that these multicore structures are polycrystalline. Their morphology is presented in the TEM micrographs and the single-core (NP15) and multicore (NF60) nature of the samples can be confirmed (Figure 2d). Hydrodynamic sizes estimated by DLS photo correlation spectroscopy (Figure 2b) indicate a certain aggregation degree of the particles in colloidal form (169 nm (PDI = 0.23) to 509 nm (PDI = 0.46) for NP15 and NF60, respectively).

Samples were further characterized by FTIR and TGA (Figure 2e,f). The FTIR spectra showed similar vibration bands for both samples. The Fe-O bonds vibration of the MIONPs generate a wide band between 480–650 cm^−1^, characteristic of magnetite and/or maghemite. Between 1000–1100 cm^−1^, a small broad band attributed to the C–O–C vibrations of EG and DEG chains can be seen. Furthermore, the band at ≈1600 cm^−1^ can be seen and corresponds to the C=O stretching vibrations corresponding to iron-coordinated carboxylates [37]. The band at 3430 cm^-1^ can be attributed to the coordinated –OH groups at the surface of the particles or the water molecules, specifically the stretching vibration of O–H [18].

Quantification of the remaining polyol molecules attached to the surface of the MIONPs was analyzed by TGA measurements. Both samples (NP15 and NF60) presented a similar behavior with two substantial and defined weight losses. As it can be seen in Figure 2f, NP15 and NF60 presented a first loss below 200 °C of 5% and 6%, respectively, attributed to the evaporation of the residual water molecules on the samples. At higher temperatures, there is a second loss step at 200–600 °C, 12% for EG and 10% for DEG, likely corresponding to the polyols’ decomposition. At around 600 °C, there is a slight weight loss that can attributed to the sintering of the particles.

The textural characteristics of NP15 and NF60 are shown in Table 2 and Figure S2 (Supplementary Information). The complex multicore structure of NF60 is reflected in a larger surface area (120 m^2^/g for NF60 vs. 60 m^2^/g for NP15). The pore size distribution in Figure S2 only shows porosity associated with particle–particle aggregation (around 7 nm for NP15 and 20 nm for NF60). This result anticipates a better adsorption capacity for NF60.

### 3.3. Magnetic Properties

**Figure 3:**
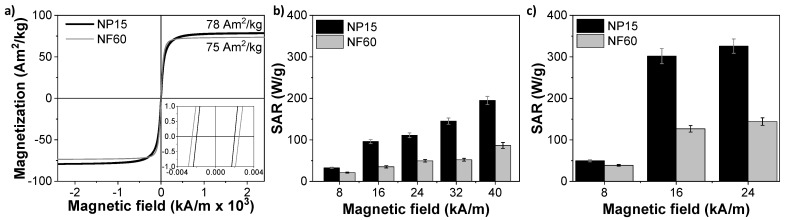
Magnetic properties of NP15 and NF60. (**a**) Magnetic hysteresis loop at room temperature of the particles (inset: low field range magnification) and SAR (W/g_Fe_) values of the samples exposed to alternating magnetic field (AMF) vs. field intensity at two frequencies: (**b**) 100 kHz and (**c**) 200 kHz.

The different microstructures confer different magnetic properties to the two selected samples. Figure 3a shows the magnetization loops obtained under quasistatic magnetic fields. It can be observed that although both samples reach magnetization values similar to magnetite [38], sample NF60 presents higher susceptibility at room temperature with a larger coercive field (H_C_^NF60^ = 2.4 kA/m (30 Oe) vs. H_C_^NP15^ = 2.0 kA/m (25 Oe)). Surprisingly, despite their differences in size and nanocrystalline structure, both samples present similar coercivity. Very low coercivity values suggest a superparamagnetic-like behavior at room temperature. Thus, below a critical diameter, which has been previously measured for magnetite particles at around 20 nm [39,40,41], thermal effects are strong enough to spontaneously demagnetize a previously saturated sample. This critical diameter is well above the particle size measured for NP15 and also for NF60, taking into account the core size (7 nm). This result indicates that the cores within NF60 are not working cooperatively, probably due to the fact that they are coming from a disordered aggregation, although certain dipolar interactions are not discarded, leading to a coercivity similar to that for NP15 [42].

The potential of the samples as self-heating catalysts was evaluated by determining their Specific Absorption Rate (SAR) at different AMF conditions. This parameter quantifies the amount of energy that is absorbed from radio-wave radiation and transformed into heat by the MIONPs. The heating curves indicated that sample NP15 is a better nanoheater, reaching SAR values up to 326 W/g_Fe_ for AMF conditions of 200 kHz and 24 kA/m. At similar conditions, the SAR of NF60 sample was limited to 144 W/g_Fe_. Figure 3b,c display the SAR dependence with the maximum applied field for AMF of 100 kHz and 200 kHz, respectively. At 100 kHz, both samples present an almost linear growth of SAR with the intensity of the applied field, while at a higher frequency, the SAR displays a drastic increment at 16 kA/m.

The weaker heating performance of NF60 with respect to NP15 might be explained by the small size of its cores. The crystal size of this sample is 7 nm. Thus, despite the size of the aggregate being 60 nm, the magnetic response of the nanostructure is generated by the individual cores. In contrast to exchange-coupled magnetic nanoflowers [43], when magnetic cores are randomly aggregated and there is not an interfacial coupling between neighboring nanocrystals, the intra-aggregate dipolar interactions may frustrate the magnetization of the system and the amount of heat dissipated in the presence of AMF results reduced [42,44].

It is interesting to observe that when both systems are well-blocked (200 kHz), it is necessary to apply a maximum applied field larger than 8 kA/m to obtain a significant amount of heat. Over 16 kA/m, little increments of the SAR values were observed in both cases. Thus, 200 kHz and 16 kA/m were selected as ideal AMF conditions to balance the heat dissipation and energy efficiency for the degradation process. The thermal curves obtained at these AMF conditions (Figure S3) indicated that to heat the reactor up to 90 °C, it was necessary to increase the MIONPs concentration up to 2 g_MIONPs_/L and 3 g_MIONPs_/L for NP15 and NF60, respectively. With the magnetic conditions selected, NP15 and NF60 (1 g_MIONPs_/L) can heat solutions up to just 50 and 35 °C, respectively.

### 3.4. Thermal Degradation and Magnetic Induction Heating-Assisted Degradation

**Figure 4:**
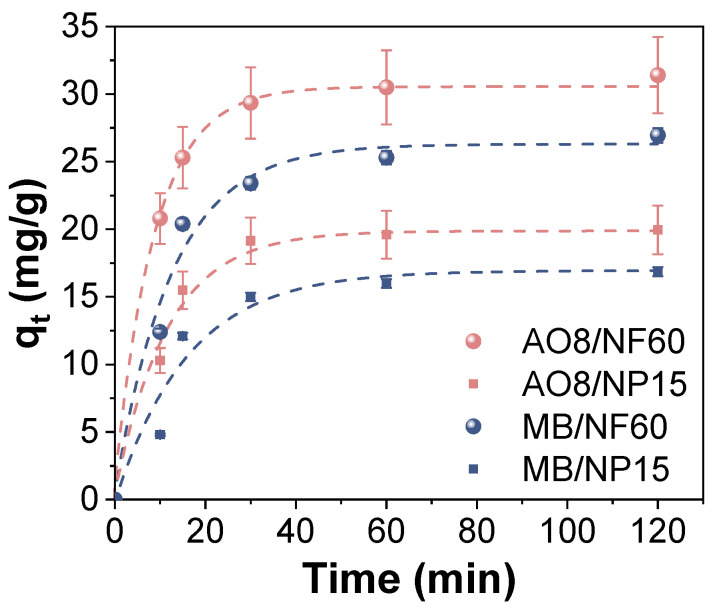
Effect of time on the acid orange 8 (orange labels) and methylene blue (blue labels) adsorption capacity using NP15 (squares) and NF60 (spheres) at room temperature and pH 3.5. Lines correspond to pseudo-first-order fitting.

The adsorption kinetics of the AO8 and MB using NP15 and NF60 were studied first. The adsorption capacity at equilibrium was obtained for different pH values, see Figure S4. For AO8, the pH where the adsorption process was most efficient was 3.5, agreeing with other reported results, while MB increased with pH but not in a significant manner. Therefore, and for comparison purposes, a pH value of 3.5 was selected to analyze the effect of adsorption time and to determine the time required to saturate the samples. Figure 4 shows the kinetics obtained for both samples in AO8 and MB solutions. A pseudo-first-order kinetic model was fitted to the experimental data showing good agreement with all of them (R^2^ > 0.98). This kinetic model usually fits well to this kind of environmental processes and indicates that chemisorption is the most likely mechanism of adsorption with the surface adsorption as a rate-limiting step [18]. Moreover, as it can be seen at 60 min of adsorption, all the samples reached the equilibrium. NF60 is a more efficient adsorbent for AO8 and MB, which can be attributed to the better textural characteristics of this sample, as mentioned before (Table 2 and Figure S2). In light of these results, degradation studies were performed after 2 h of agitation to ensure that the system had reached the equilibrium.

**Figure 5:**
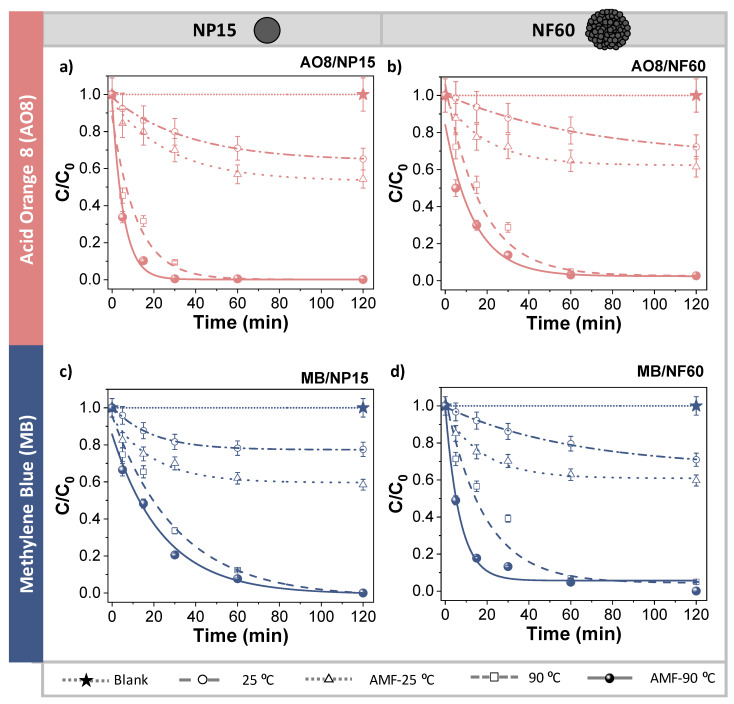
Comparison of the advanced oxidation of organic dyes. Decolorization at 25 °C, 90 °C performed in a thermal reactor, and in the presence of an alternating magnetic field of (**a**) AO8 with NP15, (**b**) AO8 with NF60, (**c**) MB with NP15, and (**d**) MB with NF60. The star symbol represents the blank sample: 500 ppm of MB or AO8, pH 3.5, H_2_O_2_ 0.3 M, 90 °C, 2 h. Lines are included as guides to the eye.

Once the adsorption capacity of the samples and the time to reach the adsorption equilibrium were determined, degradation studies as a function of time were carried out at pH = 3.5 after the addition of a specific amount of H_2_O_2_ (0.3 M). This amount of H_2_O_2_ and a pH of 3.5 are the typically reported values favorable for dye degradation [20,45]. Additionally, the selected parameters ensure that at ambient conditions, the organic dyes will not be 100% degraded, and in this way, it is possible to compare the effect of the temperature. Once the H_2_O_2_ was added, small aliquots of the mixture were withdrawn at 5, 15, 30, 60, and 120 min. Figure 5 compares the decolorization yields vs. reaction time at different experimental temperatures with the effect of the AMF.

Both MIONPs showed low efficiencies at ambient temperature (25 °C), but the aqueous solutions were fully decolorized when performing the degradation at 90 °C in the thermal reactor. Analyzing the leaching of the MIONPs at this temperature by ICP-OES, a loss of approximately 4% *w*/*w* of MIONPs into the solutions was observed (~40 ppm of total Fe in the supernatant). Therefore, the full decolorization of the samples at 90 °C may be credited to the contribution of the degradation of the pollutants at the catalysts surface (heterogeneous) and in the media, thanks to the Fe^2+^ ions in solution (homogeneous).

To just analyze the effect of the AMF on the surface catalyst, a time-dependent degradation experiment (AMF-25 °C) was conducted under an AMF of 16 kA/m and 200 kHz starting from the sample at room temperature and at 1 g_MIONPs_/L. After only 2 h, the temperature of the media increased up to 50 °C and 35 °C for NP15 and NF60, respectively. However, the nanoparticle surface temperature is expected to increase immediately after the AMF is applied [46]. As it can be observed in Figure 5, the samples showed an increase of the decolorization yield, which may be attributed to the reaction at the surface of the particles. Even though the whole mixture was slightly heated up, the particle surface was already at their maximum temperature, accelerating and stimulating the formation of the free radicals that degrade the organic pollutants, doubling the discoloration yields at 30 min compared to those obtained at 25 °C for NF60 sample and both dyes. In other words, magnetic induction heating significantly improves the decolorization of AO8 and MB with small changes of media temperature (10 °C).

To prove that it is possible to fully decolorize the samples using magnetic induction heating-assisted processes, the same AMF was applied to the particles to reach 90 °C before adding the H_2_O_2_ (the same experimental temperature as in thermal reactor). For this purpose, the MIONPs concentration was settled at 2 g_MIONPs_/L for NP15 and 3 g_MIONPs_/L for NF60 as these are the minimum values to obtain such temperatures in the medium. As it can be observed in Figure 5, magnetic induction heating reached the 100% decolorization yields observed for thermal reactor heating. However, at this temperature (90 °C), a certain iron leaching was detected in the form of Fe^2+^. Thus, by using MIONPs under an AMF, it was possible to improve the decolorization of the samples (AMF-25 °C), and to reach complete decolorization, but the maximum local temperature still needs to be optimized to avoid iron leaching. Further work is in progress to differentiate homogeneous catalysis due to dissolved Fe^2+^ and heterogeneous catalysis at the MIONPs surface.

The data obtained from the decolorization of AO8 and MB as a function of time were analyzed and found to fit well to the pseudo-first-order kinetics. These results are in good agreement with previous studies on the degradation of organic pollutants [47,48,49]. In Table 3, a summary of the obtained decolorization yields plus the kinetic constants is presented with their respective correlation coefficients. Assuming that the hydroxyl radical concentration is constant during the whole process, the organic dyes (OD) removal rate (r) can be obtained from Equation (1) using k_app_ as the constant global apparent rate constant for OD decolorization:

r = −1/*a* (d[OD]/dt) = −k_1_[OH•]^α^.[OD] = k_app_[OD] (1)

The half-life times (t1/2, Table 3) were obtained from the experimental data (Figure 5) and compared with the theoretical value for the full decolorization experiment at 90 °C under AMF conditions. The theoretical t1/2 closely approximated the measured experimentally.

The decolorization yields of AO8 were much higher than for MB when using NP15 and NF60. This may be attributed to either the different chemical nature of the dye molecules and/or the fact that AO8 is an anionic dye while MB is a cationic dye, or both. In the latter case, a strong interaction of the anionic dye (AO8) with the iron oxide at pH = 3.5 can be expected, in which the surface of MIONPs is positively charged. Interestingly, NF60 showed similar yields to the obtained with NP15 in the experiments at ambient temperature, which agrees with the better adsorptive capabilities of the former vs. the latter due to its multicore structure. Furthermore, in the experiments performed under the AMF with no previous heating (AMF-25 °C), both NP15 and NF60 showed similar decolorization yields. It should be considered that NF60 heats up the solution to a lower temperature (35 °C) than NP15 (50 °C). Therefore, NF60 is apparently a more efficient system for the heterogeneous catalytic degradation of AO8 and MB. In general, the heat from the particles’ surface and the dissipated heat in the media are both considerable parameters for the improvement of either heterogeneous or homogeneous reaction.

**Figure 6:**
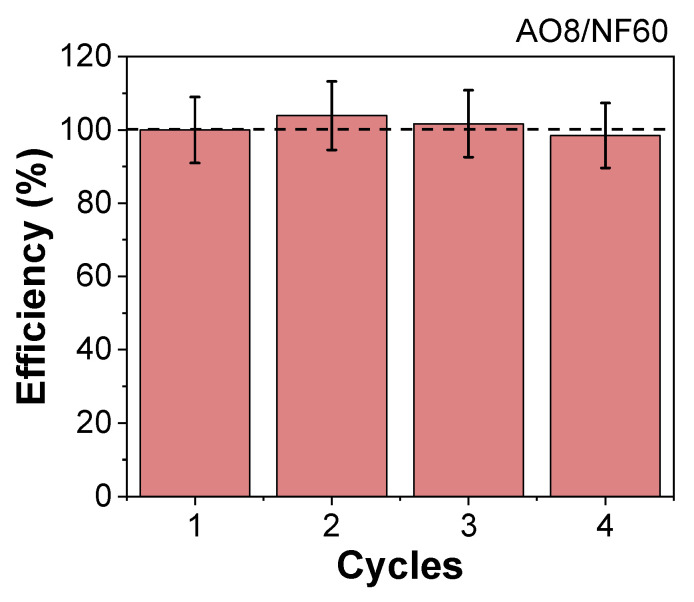
Reuse cycles of NF60 in the magnetic induced degradation of AO8 (3 g/L of NF60, 500 ppm of AO8, 0.6 M H_2_O_2_, 1 h, 16 kA/m, 200 kHz).

To date, the full potential of MIONPs as catalysts in different processes has not been completely evidenced. By taking advantage of this material’s heating capacities, it is possible to provide more efficient heating, leading to the improvement of different temperature dependent reactions. As shown in the presented results, by subjecting these particles to an AMF, it is possible to increase the surface temperatures while quickly raising the degradation efficiencies without having to wait for the medium to be fully heated. Therefore, it was possible to endorse the significance of these results by comparing them with the latest related research focused on degradation of AO8 and MB with magnetic nanomaterials. Among these works, and to the best of the authors’ knowledge, there is not a single one improving the efficiency of the AO8 degradation with magnetic induction heating of MIONPs while for MB, there is a single study where the improvement of the degradation with MIONPs obtained by electrochemistry is reported [20]. Another single study shows that the efficiency of degradation can be enhanced for emerging pollutants too, reaching 100% yields in shorter times (15 min) in solutions of 5 ppm of persistent antibiotic sulfamethoxazole, 25 mg/L of H_2_O_2_, and 1 g/L of magnetite catalyst. [21]. As this is an emerging subject, there are already some studies considering other approaches of magnetically induced remediation processes using different pollutants and materials. Phenrat C et al. showed that the degradation rate constants of the chlorinated dense non-aqueous phase liquid can be increased up to ≈60 times with magnetic induction heating of a nanoscale zerovalent iron catalyst (87 °C). In addition, Chen et al., using an induction reactor with a carbon-coated Fe^s^ magnetic catalyst, showed that the heating coming from this material can rise temperatures in a much efficient manner (up to 60 °C) allowing higher yields in the catalytic wet peroxide oxidation of direct blue, direct violet, and direct scarlet [50]. All of the mentioned studies, like the present one, were able to achieve a 100% degradation of a specific pollutant, but the most remarkable outcome is that the transference of energy occurs directly from the catalyst interface rather than the aqueous phase. This kind of process can be catalogued as more efficient speaking from a thermal point of view [16]. Moreover, the degradation of AO8 using the most efficient catalyst (NF60) showed a 100% efficiency for up to four cycles of reuse (Figure 6).

### 3.5. Proposed Reaction Mechanism

**Figure 7:**
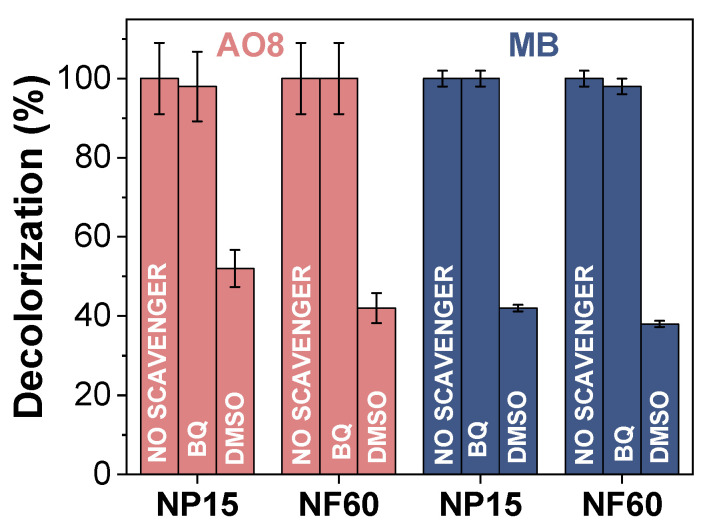
Effect of two different scavengers (DMSO: Dimethyl sulfoxide, BQ: Benzoquinone) on the degradation of AO8 and MB using NP15 and NF60. (Experimental conditions: 500 ppm of MB or AO8, 1 g/L of MIONPs, pH 3.5, H_2_O_2_ 0.3 M, 2 h, room temperature.

In advanced oxidation processes for the degradation of organic pollutants, one important parameter to consider is the reactive oxygen species (ROS) that should be formed in order to transform the organic matter into harmless products. Among these, ROS are the hydroxyl radical (OH•), hydroxyl ion (OH^−^), and superoxide anion radical (O_2_^−^•), among others. These ROS can be generated as necessary intermediates of metal catalyzed oxidation reactions. Here, the ROS were formed by the reaction between MNIOPs and the added H_2_O_2_. To elucidate whether the degradation is occurring by one or another ROS, the reaction at 90 ºC was performed in the presence of two different scavengers: DMSO and BQ. As showed in Figure 7, the decolorization efficiencies of both AO8 and MB were greatly suppressed in the presence of DMSO (30 mM), which is a common and effective OH• scavenger. On the other hand, BQ did not suppressed the degradation process, indicating that O_2_^−^• is not a degradation intermediate [51]. With this result in consideration, it is possible to assume that a Fenton mechanism is taking place in the degradation of AO8 and MB with NP15 and NF60 confirming the presence of interfacial Fe^2+^ in the MIONPs surface given by its magnetite-maghemite nature (as showed by IR and X-ray diffraction, Figure 2).

## 4. Conclusions

We show here the advantage of using in-situ heating for the formation of iron oxide nanoparticles (microwave radiation) and for the catalytic degradation of organic dyes (magnetic radiowave) in comparison to conventional thermal heating, with larger temperature gradients and slower heating rates.

First, optimizing the experimental conditions and in particular the polyol media, we manage to control particle size and aggregation, leading to uniform single-core and multicore iron oxide nanostructures that can work as efficient self-heating catalysts in the degradation of acid orange 8 and methylene blue. Secondly, advanced oxidation processes using magnetic induction heating provided a more sustainable way of reaching high degradation yields with an efficient heating coming directly from the catalyst. By subjecting the particles to an alternating magnetic field, it was possible to rise the decolorization yield by 10% considering that it was not necessary to heat up the entire solution. When the AMF is ON, the temperature at the particles’ surface immediately increases to maximum possible enhancing surface reactions. Even though the 100% decolorization was achieved at 90 ºC, a 40 ppm of Fe leaching was observed, indicating that this yield might be due to the contribution of homogeneous catalysis. Therefore, another remarkable result obtained here lays on the differentiation of the surface reaction with the Fe^2+^ in solutions. Moreover, it was possible to determine that multicore nanoparticles work better than single-core nanoparticles, as the first ones can adsorb more organic molecules. NF60 show 100% efficiencies in four reusability cycles. The preliminary mechanistic analyses indicate a production of highly active OH• species generated in a Fenton mechanism by the reaction of hydrogen peroxide with the Fe^2+^ present in the iron oxide nature of the particles. Nevertheless, further analyses are needed to better understand this mechanism as the iron leaching may be interfering with the experimentation. The presented process stands as an alternative for wastewater treatment and can successfully satisfy the need of new inventive and novel materials.

## Figures and Tables

**Table 1:** Experimental conditions explored to produce single-core and multicore flower-like magnetic iron oxide nanoparticles (MIONPs). Samples selected for further experimentation are highlighted.

Sample	Solvent	Water	Heating Rate	Temp.	Structure	TEM Size
		(%)	(°C/min)	(°C)		(nm)
Ramp1	DEG	0	3.75	220	Single core	5.8 ± 1.0
Ramp2	DEG	0	7.30	220	Single core	6.4 ± 1.0
Ramp3	DEG	0	14.6	220	Single core	7 ± 1
NP15	DEG	3.7	3.75	170	Single core	15 ± 3
Solv1	TREG	3.7	3.75	170	Single core	<5
Solv2	TEG	3.7	3.75	170	Single core	<5
NF60	EG	3.7	3.75	170	Multicore	60 ± 8

**Table 2:** MIONPs textural parameters.

Parameter	NP15	NF60
BET area (m^2^/g)	60.1 ± 0.2	118.3 ± 2.1
Pore volume (cm^3^/g)	0.14	0.19

**Table 3:** Decolorization yields (DY) and pseudo-first-order kinetic results for AO8 and MB degradation by NP15 and NF60 (N/C: Not calculated due to non-isothermal conditions).

MIONPs		Dye	T_i_^Ϯ^	T_f_^ϮϮ^	AMF *	DY	k_app1_ **	R^2^ **	t_1/2_ **
g/L			°C	°C		%	(10^−3^) min^−1^		min
NP15	1	AO8	25	25	OFF	31	7.10	0.9532	98
NP15	1	AO8	25	50	ON	46	N/C	N/C	N/C
NP15	1	AO8	90	90	OFF	100	69.9	0.8097	9.9
NP15	2	AO8	90	90	ON	100	106	0.8804	6.5
NF60	1	AO8	25	25	OFF	33	4.40	0.9999	158
NF60	1	AO8	25	35	ON	40	N/C	N/C	N/C
NF60	1	AO8	90	90	OFF	100	23.1	0.9143	30
NF60	3	AO8	90	90	ON	100	61.6	0.9295	11.2
NP15	1	MB	25	25	OFF	22	6.80	0.9999	102
NP15	1	MB	25	50	ON	41	N/C	N/C	N/C
NP15	1	MB	90	90	OFF	100	34.5	0.9999	20
NP15	2	MB	90	90	ON	100	46.1	0.9341	15
NF60	1	MB	25	25	OFF	29	3.70	0.9659	157
NF60	1	MB	25	35	ON	40	N/C	N/C	N/C
NF60	1	MB	90	90	OFF	100	35.8	0.9134	19
NF60	3	MB	90	90	ON	100	65.4	0.8626	10.6

^Ϯ^ T_i_ = Initial reaction temperature. ^ϮϮ^ T_f_ = Final reaction temperature (after 2 h). * AMF = alternating magnetic field, 16 kA/m and 200 kHz. ** Corresponding to pseudo first-order kinetics.

## Data Availability

Data is contained within the article and Supplementary Materials.

## Supplementary Materials

The following are available online at https://www.mdpi.com/article/10.3390/nano11041052/s1, Figure S1: TEM micrographs with their respective particle size distribution for Ramp1, Ramp2 and Ramp3, Figure S2 N_2_ adsorption-desorption isotherms of NP15 and NF60 with the pore size distributions, Figure S3 Temperature curves obtained at 16 kA/m, 200 kHz and 1 g/L of NP15 and NF60. Figure S4 Adsorption capacities of AO8 and MB over NP15 at different pH values (10 mg of NP15, 10 mL of AO8 500 ppm, 2 h).
