# Supplementary material for: Engineering Iron Oxide Nanocatalysts by a Microwave-Assisted Polyol Method for the Magnetically Induced Degradation of Organic Pollutants
Source: Nanomaterials (Basel). 2021 Apr 20;11(4):1052. doi: 10.3390/nano11041052 (PMC8072590; doi:10.3390/nano11041052)
Supplement: Supplementary file 1 [file nanomaterials-11-01052-s001.zip › nanomaterials-1188998-supplementary.pdf]

nanomaterials

Supporting Information

Article

Engineering Iron Oxide Nanocatalysts by a Microwave-Assisted Polyol Method for the Magnetically Induced Degradation of Organic Pollutants

Alvaro Gallo-Cordova [1,*], Sabino Veintemillas-Verdaguer [1], Pedro Tartaj [1], Eva Mazarío [2], María del Puerto Morales [1] and Jesús G. Ovejero [1,*]

[1] Instituto de Ciencia de Materiales de Madrid, ICMM/CSIC, C/Sor Juana Inés de la Cruz 3, 28049 Madrid, Spain; sabino@icmm.csic.es (S.V.V.); ptartaj@icmm.csic.es (P.T.); puerto@icmm.csic.es (M.P.M.)
[2] Departamento de Química Física Aplicada, Facultad de Ciencias, Universidad Autónoma de Madrid, C/Francisco Tomás y Valiente, 7, Cantoblanco, 28049 Madrid, Spain; eva.mazario@uam.es
* Correspondence: alvaro.gallo@csic.es (A.G.C.); jesus.g.ovejero@csic.es (J.G.O.); Tel.: +34 913 349 000 ext. 131268 (A.G.C. and J.G.O.)

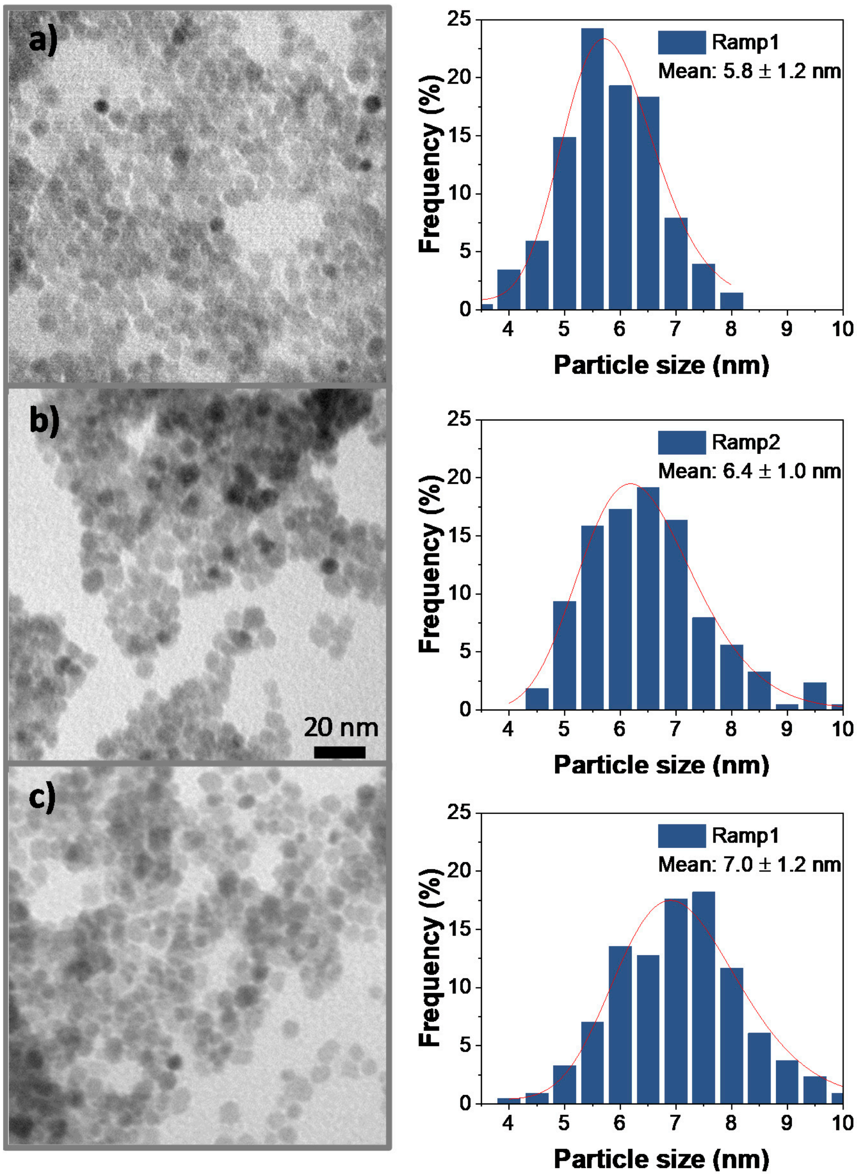

Figure S1. TEM micrographs with their respective particle size distribution of a) Ramp1 (3.75 °C/min), b) Ramp2 (7.3 °C/min) and c) Ramp3 (14.6 °C/min).

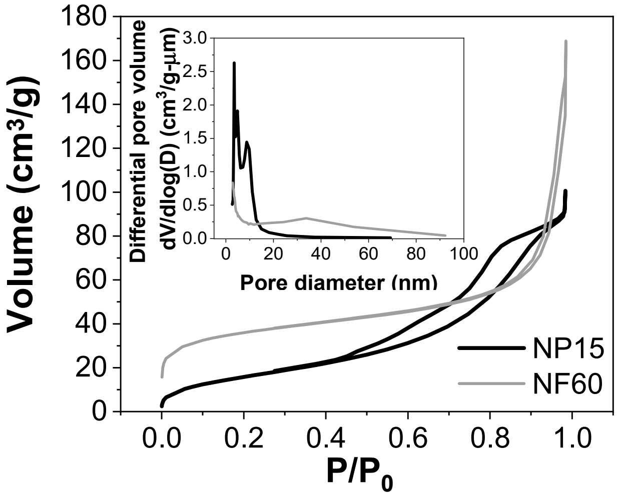

Figure S2 N_2 adsorption desorption isotherms of NP15 and NF60. Inset: pore size distribution.

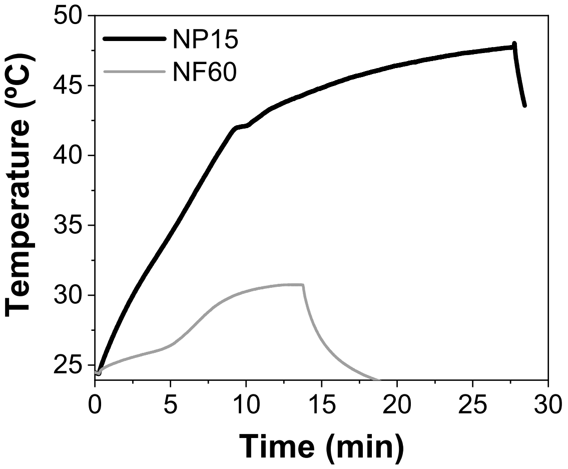

Figure S3 Temperature curves obtained at 16 kA/m, 200 kHz and 1 g/L of NP15 and NF60.

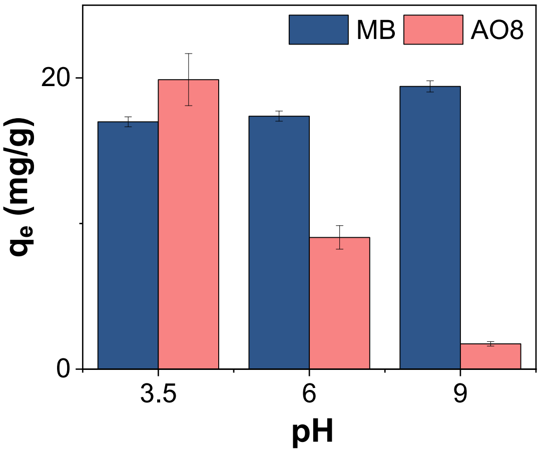

Figure S4 Adsorption capacities of AO8 and MB over NP15 at different pH values (10 mg of NP15, 10 mL of AO8 500 ppm, 2 h).
